# Structural stigma and alcohol use among sexual and gender minority adults: A systematic review

**DOI:** 10.1016/j.dadr.2023.100185

**Published:** 2023-08-18

**Authors:** Sarah S. Zollweg, Joseph A. Belloir, Laurie A. Drabble, Bethany Everett, Jacquelyn Y. Taylor, Tonda L. Hughes

**Affiliations:** a Columbia University School of Nursing, 560 W. 168th St., New York, NY, 10032, USA; b San Jose State University College of Health and Human Sciences, One Washington Square, San Jose, CA, 95192, USA; c Alcohol Research Group, Public Health Institute, 6001 Shellmound St., #450, Emeryville, CA, 94608, USA; d University of Utah, Department of Sociology, 380 S. 1530 E, Salt Lake City, UT, 84112, USA; e National Clinician Scholars Program, David Geffen School of Medicine, University of California, Los Angeles, CA, 1100 Glendon Ave, Suite 900, Los Angeles, CA 90024

**Keywords:** Alcohol, Sexual and gender minorities, LGBTQ, Structural stigma

## Abstract

**Background:**

Sexual and gender minority (SGM) people are more likely than their cisgender, heterosexual counterparts to report negative alcohol-related outcomes. Although the association between individual- and interpersonal-level minority stressors and negative alcohol-related outcomes among SGM people is well-established, structural-level minority stressors are understudied. This systematic review examined structural-level stigma and alcohol-related outcomes among SGM people to inform future research, interventions, and policy.

**Methods:**

We used five electronic databases to search for studies published between January 2010 and May 2022 that examined associations between structural stigma and alcohol use among SGM adults in the United States. Peer-reviewed, quantitative studies available in English were included. We conducted quality appraisal using the Joanna Briggs Institute checklist.

**Results:**

The final sample included 11 studies. Overall, there was moderate to strong support for a positive association between structural stigma and negative alcohol-related outcomes among SGM people, with differences by gender, sexual identity, race, and ethnicity. All studies used cross-sectional designs, and nearly half utilized non-probability samples. Transgender and nonbinary people, SGM people of color, and sexual identity subgroups beyond gay, lesbian, and heterosexual were underrepresented. Structural stigma was most commonly measured as a state-level index. Alcohol measures were heterogeneous. Multilevel stigma and resiliency factors were understudied.

**Conclusions:**

Addressing structural stigma is critical in reducing negative alcohol-related outcomes and inequities among SGM people. Research is needed that includes probability samples, longitudinal designs, and samples that reflect the diversity of SGM people. Future studies should examine the influence of multilevel stigma and resiliency factors on alcohol-related outcomes.

## Highlights

• Structural stigma is positively associated with negative alcohol-related outcomes.
• There are differences by gender, race, ethnicity, and sexual identity.
• Most studies are cross-sectional and used non-probability samples.
• Gender minority people and many sexual identity subgroups are underrepresented.
• Sexual and gender minority people of color are underrepresented.

## 1 Introduction

Excessive alcohol use is a major public health issue in the United States (U.S.), contributing annually to more than 140,000 deaths and nearly 3.6 million years of lost life between 2015 and 2019 Centers for Disease Control and Prevention (2022). Risk for negative alcohol-related outcomes differs by sexual identity (how an individual identifies sexually or romantically, e.g., lesbian, gay, bisexual, heterosexual) and gender identity (an individual's experience of gender, e.g., man, woman, nonbinary, agender, or elsewhere along a gender spectrum). Sexual minority (e.g., lesbian, gay, bisexual) and gender minority (e.g., transgender, nonbinary) people have higher prevalence of alcohol use and poorer alcohol-related health outcomes than their cisgender, heterosexual counterparts (Drabble et al., 2021; Hughto et al., 2021; Krueger et al., 2020; Schuler et al., 2018). For example, in analyses of the National Epidemiologic Survey on Alcohol and Related Conditions III, sexual minority people had 1.6–1.9 times higher odds of past-12-month alcohol use disorder (AUD) and 1.7–2.4 times higher odds of lifetime AUD than their heterosexual counterparts (Kerridge et al., 2017). This disparity was more pronounced among women; sexual minority women (SMW) had 2.1–2.5 times higher odds of past-12-month AUD and 2.2–2.7 times higher odds of lifetime AUD than heterosexual women (Kerridge et al., 2017).

Alcohol outcomes also differ within the overall SGM population. For example, in the 2013–2014 National Health Interview Survey, compared with their heterosexual counterparts, bisexual men were 3.15 times more likely, lesbian women were 2.62 times more likely, and bisexual women were 2.07 times more likely to report heavy drinking (Gonzales et al., 2016). In the 2014–2017 Behavioral Risk Factor Surveillance System (BRFSS), compared to cisgender women, “gender nonconforming” individuals had 2.09 times higher odds of heavy drinking and 1.94 times higher odds of binge drinking, and “male to female” transgender individuals had 1.88 times higher odds of binge drinking (Azagba et al., 2019). Further, in a national study of insurance claims data, the prevalence of AUD among transgender adults was 2.75 times higher than among cisgender adults (Hughto et al., 2021). There are also differences by race and ethnicity, particularly among SMW (Schuler et al., 2020). For example, compared to heterosexual counterparts of the same race/ethnicity, there was a greater disparity in heavy episodic drinking risk among Hispanic SMW (adjusted risk ratio = 1.55–1.68) and Black SMW (adjusted risk ratio = 1.38–1.56) than among White SMW (adjusted risk ratio = 1.0–1.23; Schuler et al., 2020).

Minority stress, i.e., exposure to stressors such as discrimination related to stigmatized minority status (e.g., sexual or gender identity, race, ethnicity; Brooks, 1981; Meyer, 2003), is the predominant explanatory framework used to understand SGM-related alcohol-use disparities. The minority stress model illustrates how distal minority stressors (e.g., experiences of discrimination) lead to more proximal internalizations of social stigma (e.g., internalized stigma, or negative feelings about one's sexual identity; Meyer, 2003). Minority stress wears on individuals and is associated with numerous poor physical, mental, and behavioral health outcomes among sexual and gender minority (SGM) people (Frost et al., 2015; Lewis et al., 2016, 2017). Further, SGM people with multiple marginalized identities may experience additional stressors, such as racism, that compound risk (Bowleg, 2008, 2012; Meyer, 2010).

The minority stress model focuses on minority stressors at the individual and interpersonal levels, which are well established in the literature as contributing factors to alcohol risk among SGM people. For example, internalized homophobia (Felner et al., 2020; Hequembourg and Dearing, 2013; Hughes et al., 2020), stigma consciousness (Felner et al., 2020; Lewis et al., 2017), family rejection (Everett et al., 2021; Hughes et al., 2020), and discrimination based on sexual orientation or gender identity (Hughes et al., 2016; Kcomt et al., 2020; McCabe et al., 2010, 2019; Wilson et al., 2016) have each been associated with poor alcohol-related outcomes among SGM people. However, despite evidence of harms of SGM stigma at the individual and interpersonal levels, there has been relatively little attention to stigma at the structural level (Hatzenbuehler, 2016; Hughes et al., 2020).

Some researchers more broadly interpret minority stress theory to include stigma at the structural level (Hatzenbuehler, 2016; Hatzenbuehler and Pachankis, 2016). Structural stigma is defined as “societal-level conditions, cultural norms, and institutional policies that constrain opportunities, resources, and well-being of the stigmatized” (Hatzenbuehler and Link, 2014, p. 2). Examples are policies that permit discrimination in public accommodations (e.g., refusing to provide services to SGM people) or that make it difficult for same-sex couples to adopt a child. More specific examples include “Don't Say Gay” bills, which ban discussion of sexual orientation or gender identity in public school classrooms and permit parents to sue teachers and schools for perceived violations, and bans on gender-affirming care for transgender people of all ages, including adults (Goldberg, 2023; Movement Advancement Project, 2023a). Structural stigma has primarily been conceptualized and measured via policy analysis or aggregated measures of social attitudes (Hatzenbuehler, 2016). To adequately measure stigma, it is important to differentiate structural-level factors from individual- or interpersonal-level factors. For example, absence of a state-level employment nondiscrimination law is a structural-level factor, being fired from a job due to sexual or gender identity is an interpersonal-level factor, and internalizing that experience by taking on a negative view of one's SGM identity is an individual-level factor.

Current research on minority stress and alcohol use among SGM people primarily focuses on the individual and interpersonal levels, and although there is a growing body of research on structural stigma and poor health outcomes among SGM people (Hatzenbuehler, 2014, 2016, 2018), few studies focus explicitly on alcohol-related outcomes. This research gap limits understanding and effective interventions to reduce inequities in alcohol-related outcomes among SGM people (Hatzenbuehler, 2016; Krieger, 2014). Therefore, the objective of this review was to identify, evaluate, and synthesize recent literature on structural stigma and alcohol-related outcomes among SGM adults to better inform multilevel interventions aimed at alleviating alcohol-related inequities among SGM people.

## 2 Methods

### 2.1 Literature search

This review followed the Preferred Reporting Items for Systematic Review and Meta-Analysis (PRISMA) guidelines (Page et al., 2021). Although the research team developed the protocol a priori, the review was not preregistered. We performed a comprehensive search of five electronic databases, i.e., PubMed, PsycInfo, the Cumulative Index to Nursing and Allied Health Literature (CINAHL), Embase, and LGTBQ+ Source, in May 2022. Searches included keywords and database-specific subject headings for SGM people (population), structural stigma (exposure), and alcohol use (outcome). See Appendix A for the full PubMed search strategy.

### 2.2 Study selection

**Fig. 1:**
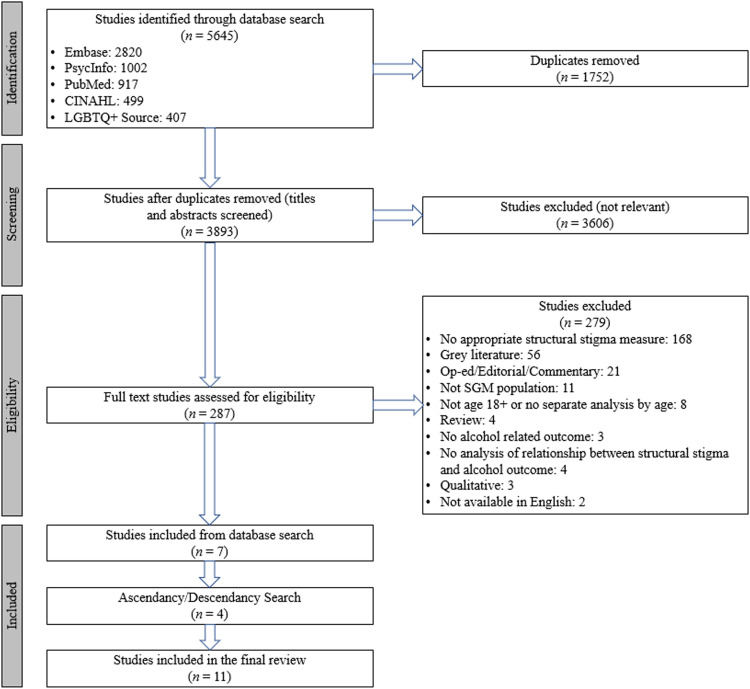
PRISMA Flow Diagram. (For interpretation of the references to color in this figure legend, the reader is referred to the web version of this article.)

Identified articles were uploaded to the EndNote citation manager to manually remove duplicates, then uploaded to Covidence (an online systematic review tool). Two authors (S.S.Z. and J.A.B) screened titles and abstracts and excluded articles that were not relevant. This process continued through full text review of remaining articles. Consensus was reached on all disagreements after discussion. Initial disagreements primarily related to whether measures of structural stigma fit the research team's predetermined definition and criteria of an objective structural stigma measure, meaning that the measure had to objectively reflect the sociopolitical environment rather than participants’ subjective experiences of structural stigma. After completion of the full text review, we performed ascendancy and descendancy searches (i.e., a search of all studies cited by each included article and a search of all articles that cited each article, respectively) to identify additional relevant articles. See Fig. 1 for a flowchart of the search process.

#### 2.2.1 Inclusion and exclusion

To meet inclusion criteria, articles had to report findings from peer-reviewed studies published in English between January 1, 2010, and May 18, 2022. Included articles quantitatively examined the relationship between structural stigma and alcohol-related outcomes among SGM adults in the U.S. using an objective measure of structural stigma. For example, measures such as presence or absence of state-level policies that are discriminatory toward SGM people met inclusion criteria. In contrast, measures evaluating participants’ perceptions of their exposure to structural stigma did not fit the predetermined definition of an objective measure. Because sociopolitical climate and attitudes toward SGM people vary widely between countries, studies outside the U.S. were excluded. Additionally, qualitative studies, literature reviews, case studies and case series, unpublished dissertations, op-eds and other opinion pieces, and editorials were excluded.

We chose 2010 as the earliest date for our search because this marked the beginning of a rapid succession of changes in the sociopolitical environment for SGM people, making earlier studies potentially less relevant to the current context. For example, in 2010, Don't Ask, Don't Tell (a policy that prohibited people in the military from disclosing SGM identities) was repealed (Don't Ask, Don't Tell Repeal Act, 2010) and the Office of Disease Prevention and Health Promotion's (2010) Healthy People objectives included SGM health as a priority for the first time. In 2011, Joint Commission published a lesbian, gay, bisexual, transgender (LGBT) field guide (Tschurtz et al., 2011), and the Institute of Medicine (now the National Academy of Medicine) published a landmark report on LGBT health (Institute of Medicine, 2011). In addition to these sociopolitical changes and increasing attention to sexual and gender identity-related health disparities, 2010 also marked the emergence of early research studies focused on structural stigma and health among SGM people, with few articles on this topic prior to 2010. Specifically, the first seminal studies that included alcohol-related outcomes (among other psychiatric outcomes) related to structural stigma (i.e., on state ballot initiatives banning same-sex marriage in the U.S.) were published between December 2009 and March 2010 (Hatzenbuehler et al., 2009, 2010; Keyes et al., 2012).

### 2.3 Data extraction

Publication year, study population, location, sampling strategy, data source, study design, time frame, sample characteristics, study purpose, measures of structural stigma and alcohol use, and study findings were extracted into an excel table by the first author. Data extraction was then checked by the second author for accuracy and completeness.

### 2.4 Quality appraisal

The first author used the 8-item Joanna Briggs Institute (JBI) Critical Appraisal Checklist for Analytical Cross-Sectional Studies (Joanna Briggs Institute, 2020) to appraise quality of studies and risk of bias, and the second author confirmed these appraisals (see Table 1). Response options were “*Yes*” and “*No*.” The total number of “*Yes*” responses for each study were tallied. However, JBI checklists do not evaluate studies with overall ratings or percentage scores to avoid the implication that all items are weighed equally. Therefore, the tally of “*Yes*” responses is not intended as such.

**Table 1:** Quality appraisal of included studies.

	Were the criteria for inclusion in the sample clearly defined?	Were the study subjects and the setting described in detail?	Was the exposure measured in a valid and reliable way?	Were objective, standard criteria used for measurement of the condition?	Were confounding factors identified?	Were strategies to deal with confounding factors stated?	Were the outcomes measured in a valid and reliable way?	Was appropriate statistical analysis used?	Total
Blosnich et al. (2016)	Yes	Yes	Yes	Yes	Yes	Yes	Yes	Yes	8/8
Drabble et al. (2021)	Yes	Yes	Yes	Yes	Yes	Yes	Yes	Yes	8/8
Drabble et al. (2022)	Yes	Yes	Yes	Yes	Yes	Yes	Yes	Yes	8/8
Du Bois et al. (2018)	Yes	Yes	Yes	Yes	Yes	Yes	No	Yes	7/8
English et al. (2021)	Yes	Yes	Yes	Yes	Yes	Yes	Yes	Yes	8/8
Everett et al. (2016)	Yes	Yes	Yes	Yes	Yes	Yes	Yes	Yes	8/8
Greene et al. (2020)	Yes	No	Yes	Yes	Yes	Yes	Yes	Yes	7/8
Greene et al. (2021)	Yes	No	Yes	Yes	Yes	Yes	Yes	Yes	7/8
Hatzenbuehler et al. (2010)	Yes	Yes	Yes	Yes	Yes	Yes	Yes	Yes	8/8
Manser and Du Bois (2021)	Yes	Yes	Yes	Yes	Yes	Yes	Yes	Yes	8/8
Pachankis et al. (2014)	Yes	Yes	Yes	Yes	Yes	Yes	No	Yes	7/8

*Note*. Scoring is *Yes* and *No*. This table includes questions from the JBI Critical Appraisal Checklist for Analytical Cross-Sectional Studies (Joanna Briggs Institute, 2020).

## 3 Results

### 3.1 Study and sample characteristics

We retrieved 5645 articles through the database search. After removing 1752 duplicates, we screened 3893 titles and abstracts. At this stage we excluded 3606 irrelevant articles, resulting in 287 full text articles for review. During full text review we excluded 279 articles that did not meet criteria, yielding seven studies. We identified an additional four articles via ascendancy and descendancy searches, resulting in a final sample of 11 articles.

Table 2 includes a summary of study characteristics. All 11 studies were cross-sectional and just over half used probability samples. Studies included data from a variety of sources, including computer-assisted telephone surveys, online surveys, face-to-face computer-assisted personal interviews, and health records. One study used a sample recruited in the Chicago metropolitan area, and the remaining 10 studies used national U.S. samples or samples that represented a majority of states, with wide representation of U.S. regions. Data for all studies were collected between 2000 and 2019.

**Table 2:** Study characteristics.

Author (Year)	Population of interest	Location	Sample type	Data source	Study design	Time frame
Blosnich et al. (2016)	Transgender veterans receiving VA care	National U.S.	Non-probability	VA health records	Cross- sectional	Jan 2013 – Sep 2013
Drabble et al. (2021)	Adult U.S. population, excluding institutional settings (e.g., jails, detoxification centers)	National U.S.	Probability	National Alcohol Survey, CATI	Cross-sectional	2000, 2005, 2010, 2015
Drabble et al. (2022)	Adult SMW	National U.S.	Non-probability	Two national online panels	Cross-sectional	Summer & Fall 2019
Du Bois et al. (2018)	Transgender adults, excluding those in institutional settings (e.g., jails, detoxification centers) or those without phone access	U.S. states/territories that used the SOGI module (26 of 54 total)	Probability	BRFSS, CATI	Cross-sectional	2016
English et al. (2021)	Adult SMM	National U.S.	Non-probability	Understanding New Infections through Targeted Epidemiology Study (online survey)	Cross-sectional	2017–2018
Everett et al. (2016)	Adult SMW	Illinois, primarily Chicago	Non-probability	CHLEW study, face to face CAPI	Cross-sectional	Wave 3 (2010 – 2012)
Greene et al. (2020)	Adult U.S. population, excluding institutional settings (e.g., jails, detoxification centers) or those without phone access	U.S. states that used the SOGI module (35 of 50 total)	Probability	BRFSS, CATI	Cross-sectional	2015–2018
Greene et al. (2021)	Adult U.S. population, excluding institutional settings (e.g., jails, detoxification centers) or those without phone access	U.S. states that used the SOGI module (35 of 50 total)	Probability	BRFSS, CATI	Cross-sectional	2015–2018
Hatzenbuehler et al. (2010)	Noninstitutionalized adult LGB and heterosexual civilians	National U.S.	Probability	NESARC, face to face CAPI	Cross-sectional	Wave 1 (2001 – 2002), Wave 2 (2004 – 2005)
Manser & Du Bois (2021)	Lesbian women and gay men, excluding institutional settings (e.g., jails, detoxification centers) or those without phone access	U.S. states that used the SOGI module (27 of 50 total)	Probability	BRFSS, CATI	Cross-sectional	2017
Pachankis et al. (2014)	Young SMM enrolled as full-time university students	Four U.S. regions (Northeast, South, Midwest, West), including 24 states	Non-probability	Online survey spanning 9 consecutive days	Cross-sectional	Not reported

*Note*. SOGI = Sexual orientation and gender identity. VA = Veterans Affairs. BRFSS = Behavioral Risk Factor Surveillance System. NESARC = National Epidemiologic Survey on Alcohol and Related Conditions. CHLEW = Chicago Health and Life Experiences of Women. CAPI = Computer Assisted Personal Interview. CATI = Computer Assisted Telephone Interview. SOGI = Sexual Orientation and Gender Identity. SMW = Sexual Minority Women. SMM = Sexual Minority Men.

For sample characteristics, see Table 3. There was a wide range of sample sizes, from 119 to 863,257, with a median size of 3057. Six studies reported mean age, ranging from 34.04 to 54.8 years old. In five of the nine studies reporting race and ethnicity, samples were more than 70% non-Hispanic White, ranging from 37.2% to 81.2%, with a median of 70.92% non-Hispanic White. Two studies did not report numbers or percentages of race or ethnicity. Only two studies examined differences by race or ethnicity and no studies examined age differences.

**Table 3:** Study findings.

Author (Year)	Sample characteristics	Study purpose	Measure of structural stigma	Measure of alcohol outcome	Main findings
Blosnich et al. (2016)	*N* = 1640; age 18+ (*M* = 54.8, *SD* = 13.2); 69.3% male, 30.7% female; 100% transgender; 81.2% White, 15.1% Black, 3.8% other race; 95.3% non-Hispanic, 4.7% Hispanic	To examine whether community- and state-level LGBT equality are associated with mental health (including substance use) among transgender veterans receiving VA care	• City-level Municipality Equality Index (MEI): ○ Non-discrimination laws ○ Same-sex marriage ○ Equal benefits/ protections ○ LGBT inclusion in city services, law enforcement, hate crime statistics ○ Relationship of city leaders with LGBT community • State-level employment non-discrimination (yes/no) • State-level hate crime laws inclusive of gender identity or transgender status (yes/no)	Alcohol Abuse Disorder: ICD-9 code 303	Among transgender veterans: • Neither state-level employment non-discrimination protections nor inclusive hate crime laws were associated with AUD • Transgender veterans with AUD lived in cities with higher mean state MEI scores (i.e., greater equality) compared to those without AUD (78.4 vs 73.6, *p* = 0.02) • A one-point increase in MEI was associated with 1% increase in odds of AUD
Drabble et al. (2021)	*N* = 25,210; 34% age 18–39, 66% age 40+; 55% women, 45% men; 3.3% sexual minority, 96.7% heterosexual; 58.8% White, 18.6% Black, 18.4% Hispanic, 4.3% Other	To examine whether state-level structural stigma moderated the relationship between sexual identity and substance use	State policy environment based on Movement Advancement Project, dichotomized as full protections vs anything else:Positive laws: • Legalized same-sex marriage • Non-discrimination laws in adoption/ fostering for same-sex couples • Employment non-discrimination laws • Housing non-discrimination laws • Non-discrimination laws in public accommodations • Hate crime laws inclusive of sexual minority people Negative laws: • Explicit same-sex marriage ban • Policies allowing discrimination in adoption/fostering • State bans on cities/counties passing nondiscrimination laws • Religious exemption laws allowing discrimination based on religious/moral grounds	AUD: endorsement of 2 or more of 11 domains in the DSM-5 (includes mild, moderate, and severe AUD) High Intensity Drinking: consumption of 8+ drinks in one day at least once in past year, vs none	High-intensity drinking: • The interaction between comprehensive protections and SI was significant among men (OR = 0.31, *p* < 0.001) but not women (OR = 0.39, *p* = 0.063), i.e., full protections were associated with decreased odds of high-intensity drinking among SMM (but not among heterosexual men, heterosexual women, or SMW) • SMM had lower probability of high intensity drinking in states with comprehensive protections compared to SMM in states without (Pr = −0.11, *p* = 0.001), but SMW did not DSM-5 AUD: • There was no significant interaction between comprehensive protections and SI for men or women • There was no significant difference in probability of DSM-5 AUD among SMW or SMM in states with comprehensive protections compared to states without
Drabble et al. (2022)	*N* = 732; age 18+ (*M* = 35.45, SD = 13.45); 100% women; 59.70% lesbian, 40.30% bisexual; 37.43% White, 25.82% Black, 31.15% Latinx, 5.60% Other/Missing	To examine whether state-level structural stigma influenced substance use among SMW	State policy environment based on Movement Advancement Project, dichotomized as full protections vs anything else:Positive laws: • Non-discrimination laws in adoption/ fostering for same sex couples • Employment non-discrimination laws • Housing non-discrimination laws • Non-discrimination laws in public accommodations • Hate crime laws inclusive of sexual minority people Negative laws: • Policies allowing discrimination in adoption/fostering • State bans on cities/counties passing nondiscrimination laws • Religious exemption laws allowing discrimination based on religious/moral grounds	Heavy episodic drinking: number of days, past year, of 4+ drinks in one day (continuous)	Comprehensive policy protections were significantly associated with fewer days of heavy episodic drinking among SMW (*b* = −15.11, *p* = 0.38). In sensitivity analyses, this was significant among bisexual (*b* = −37.168, *p* < 0.01) but not lesbian women.
Du Bois et al. (2018)	*N* = 1116; age 18–80 (*M* = 42.3, SD = 18.2); 49.3% transgender women, 28.3% transgender men, 22.4% gender nonconforming; 58.9% straight, 13.0% lesbian/gay, 15.5% bisexual, 6.6% other SI, 2.2% don't know/not sure, 3.7% missing; 51.2% White, 22.0% Hispanic, 15.3% Black, 5.3% Asian, 2.7% Multiracial, 1.3% American Indian or Alaskan Native, 0.5% Native Hawaiian or Pacific Islander, 0.5% Other, 1.1% don't know/not sure/missing	To examine whether inclusive and protective state-level transgender policies were associated with physical health, mental health, health behaviors, and health care utilization	State-level gender identity policy tally based on Movement Advancement Project, incorporating 35 different law/policy types (e.g., nondiscrimination laws, protections for SGM youth); continuous variable with higher tally indicating more inclusive policy environment	Average number of alcoholic drinks per day (not specified whether past month, past year, etc.)	A one-point increase in state-level gender identity policy tally (i.e., a more inclusive policy environment) was associated with a 0.11 decrease in average number of alcoholic drinks per day
English et al. (2021)	*N* = 6916; mean age = 34.04, SD = 12.26, med = 31; 99.2% cisgender men, 0.8% transgender men; 81.1% gay, 2.4% queer, 16.5% bisexual; 80.1% White, 19.9% Black	To examine whether anti-LGBTQ policies influence SMM's psychological and behavioral health and whether there is an interaction between anti-LGBTQ policies and structural racism	Human Rights Campaign's 2018 State Equality Index based on anti-LGBTQ state policies (e.g., HIV/AIDS criminalization, permitting conversion therapy); dichotomized as low vs high equality	Alcohol Use Disorders Identification Test-Consumption (AUDIT-C) to assess heavy drinking; 3 items with 5-point scales; higher scores indicate higher levels of heavy drinking; summed scores on each item to make continuous outcome	• Among Black SMM, structural racism (*β* = 0.23, SE = 0.10, *p* = 0.01); anti-LGBTQ policies (*β* = 0.10, SE = 0.04, *p* = 0.01); and the interaction between structural racism and anti-LGBTQ policies (*β* = 0.22, SE = 0.07 *p* = 0.003) were positively associated with heavy drinking • Anti-LGBTQ policies strengthened the positive association between structural racism and heavy drinking among Black SMM • Among White SMM, neither structural racism, anti-LGBTQ policies, or the interaction between the two were associated with heavy drinking
Everett et al. (2016)	*N* = 517; age 18–79 (*M* = 40.21); 100% women; 59.18% lesbian, 16.10% mostly lesbian, 24.72% bisexual; 37.20% non-Hispanic White, 62.80% Black or Latina	To examine the impact of Illinois’ Religious Freedom Protection and Civil Union Act (legalizing civil unions) on psychosocial health in sexual minority women	State-level civil union legalization: • Before Illinois civil union bill passed • After Illinois civil union bill was signed but before enactment • After Illinois civil union bill enactment	Hazardous drinking, past 12 months: • Heavy episodic drinking (any instance of 6+ drinks per day; binary yes/no) • Intoxication (continuous 0–7, from “never” to “5 or more times a week”) • Adverse drinking consequence (continuous count variable, from 0 to 8 consequences) • Alcohol dependence symptoms (continuous count variable, from 0 to 5 symptoms)	• In the full sample, bill signing (*β* = −0.57, SE = 0.18, *p* < 0.001) and enactment (*β* = −0.33, SE = 0.14, *p* < 0.05) were associated with fewer adverse drinking consequences, but not with alcohol dependency or with intoxication; bill enactment was associated with increased heavy episodic drinking (*β* = 0.45, SE = 0.23, *p* < 0.05) • After the bill was signed, Black and Latina SMW (*β*= −0.22, SE = 0.13, *p* < 0.10) had significantly less frequent intoxication than White SMW (*β*= −0.22 + 0.42, *p* < 0.05) • There was no significant difference in dependence symptoms among Black and Latina SMW before vs after the bill was signed; White SMW interviewed after the bill was signed reported significantly more dependence symptoms compared to White SMW before the bill was signed (*β* = 0.81, SE = 0.37, *p* < 0.05) • SMW with high school education or less (*β*=−1.65, *p* < 0.05) reported significantly less heavy episodic drinking after the bill was signed vs those interviewed before the bill was signed; SMW with some college (*β*=−1.65 + 2.01, *p* < 0.05) or college degree (*β*=−1.65 + 2.04, *p* < 0.01) did not
Greene et al. (2020)	*N* = 863,257; age 18+; 56.18% women, 43.82% men; 47.87% straight women, 0.57% lesbian women, 1.03% bisexual women, 36.89% straight men, 0.81% gay men, 0.56% bisexual men; race or ethnicity not reported	To investigate whether the presence of state nondiscrimination statutes modifies the positive association between sexual identity and binge drinking	Presence state-level nondiscrimination statutes inclusive of sexual orientation in employment, housing and public accommodations vs none (binary; all included states either had all 3 nondiscrimination statutes or none), based on Human Rights Campaign State Equality Index and Movement Advancement Project (there was complete agreement between the two sources for all included states)	Binge drinking, past 30 days: One or more times of 4+ drinks for women or 5+ drinks for men, vs zero times (dichotomous)	• In states without inclusive statutes, lesbian women had 1.71 (95% CI: 1.27–2.30) times higher odds of binge drinking than straight women, whereas in states with inclusive statutes, the odds were not significantly different • In states without inclusive statutes, bisexual women had 1.83 (95% CI: 1.55–2.17) times higher odds of binge drinking than straight women, whereas in states with inclusive statutes, bisexual women only had 1.35 (95% CI: 1.13–1.60) times higher odds of binge drinking than straight women • There was no significant interaction between inclusive statutes and sexual identity for gay or bisexual men
Greene et al. (2021)	*N* = 775,581; age 18+; 56.21% women, 43.79% men; sexual identity, race, and ethnicity not reported	To investigate whether the presence of state nondiscrimination statutes modifies the association between state alcohol policy environment and binge drinking and whether this interaction differed by sexual identity	Presence state-level nondiscrimination statutes inclusive of sexual orientation in employment, housing and public accommodations vs none (binary; all included states either had all 3 nondiscrimination statutes or none), extracted from Movement Advancement Project online reports	Binge drinking, past 30 days: One or more times of 4+ drinks for women or 5+ drinks for men, vs zero times (dichotomous)	• In states with inclusive statutes, a 10-point increase in Alcohol Policy Scale (i.e., a stricter alcohol policy environment, e.g., higher alcohol taxes) was associated with 0.93 times lower odds (95% CI: 0.89–0.97, *p* = 0.0003) of binge drinking among women, regardless of sexual identity • In states without inclusive statutes, a 10-point increase in Alcohol Policy Scale was not associated with binge drinking among women • Among men, there was no association between state alcohol policy environment and binge drinking for heterosexual, gay, or bisexual men, regardless of presence of inclusive state statutes
Hatzen-buehler et al. (2010)	*N* = 34,653; age among LGB respondents 13.5% ≤25, 49.4% 26–45, 31.1% 46–64, 6.1% ≥65; age among heterosexual respondents 9.2% ≤25, 38.8% 26–45, 33.5% 46–64, 18.5% ≥65; 98.59% heterosexual, 1.67% sexual minority; 52.09% female, 47.91% male; 70.92% White, 11.58% Hispanic; 10.99% Black, 4.28% Asian, 2.22% American Indian	To examine the relationship between state marriage laws and prevalence of psychiatric disorders	State-level marriage laws: states that voted on and passed constitutional amendments in 2004–2005 defining marriage as between a man and a woman only (same-sex marriage ban) vs those that did not have such an amendment on their ballots (dichotomous)	The AUDADIS-IV: over 40 items for past 12-month DSM-IV substance abuse and dependence for alcohol and other drugs	• In states that passed a marriage ban between Waves 1 and 2, odds of AUD were 1.8 (95% CI: 1.08–3.01) times higher among LGB people at Wave 2 compared to Wave 1 • In states that did not pass a marriage ban between Waves 1 and 2, odds of AUD among LGB people did not significantly change from Wave 1 to Wave 2 • In states that passed a marriage ban between Waves 1 and 2, odds of AUD were 1.22 (95% CI: 1.09–1.35) times higher among heterosexual people at Wave 2 compared to Wave 1 (i.e., similar to LGB people, odds of AUD increased from Wave 1 to Wave 2, but at a smaller magnitude)
Manser & Du Bois (2021)	*N* = 3057; age 18+ (*M* = 42.26, SD = 16.4); 58.49% gay men, 41.51% lesbian women; 74.55% White, 8.53% Hispanic, 6.93% Black, 3.85% Multiracial, 2.38% Asian, 1.03% American Indian/Alaskan Native, 0.75% Native Hawaiian/Pacific Islander, 0.46% Other Race, 1.34%% missing	To investigate whether structural stigma is positively associated with CVD, and whether this association is mediated by substance use	State policy environment based on Movement Advancement Project, total number of state-level non-discrimination laws was used to create an ordinal index of 0 (fewest protective laws, i.e., highest structural stigma) to 5 (most protective laws, i.e., lowest structural stigma) with 5 domains of non-discrimination laws: • Employment (both public and private) • Housing • Public accommodations • Credit-lending • State employment	Binge drinking, past month: One or more instances of 4+ drinks for women or 5+ drinks for men, vs zero times (dichotomous)	• Structural stigma was positively associated with binge drinking among lesbian women (β = 0.74, SE = 0.035, *p* = 0.032) • Structural stigma was not associated with binge drinking among gay men or the combined sample of lesbian women plus gay men
Pachankis et al. (2014)	*N* = 119; age 18–25; 100% men; 94.11% gay, 15.13% bisexual, 4.20% queer, 0.84% “bisexual, equally gay and heterosexual” (note that percentages add up to >100%); 71.43% White, 8.40% Latino/Hispanic, 6.72% Asian, 5.88% Black, 3.36% Mixed Race, 2.52% Native American, 0.840% Pacific Islander, 0.840% Caribbean	To examine whether both past and current structural stigma are associated with substance use, whether rejection sensitivity mediates these associations, and whether structural stigma moderates the association between rejection sensitivity and substance use.	Structural stigma index based on z-transformed scores of: • State-level LGB policies in 2005 (past structural stigma) and 2009 (current structural stigma), summed 0–5: ○ Same-sex marriage ban ○ Employment non-discrimination ○ Inclusive hate crime laws ○ Non-discrimination and inclusive bullying laws in schools ○ Adoption non-discrimination • State-level public attitudes toward LGB-specific policies from Roper Center, mean values: ○ Adoption ○ Hate Crimes ○ Health benefits ○ Job discrimination ○ Housing discrimination ○ Marriage equality ○ Sodomy ○ Civil unions	Alcohol use vs non-use (binary) for each day of the study	• There was no significant direct association between past or current structural stigma and alcohol use • There was a significant interaction between past structural stigma and rejection sensitivity, i.e., the association between rejection sensitivity and alcohol use was stronger among participants who experienced greater structural stigma in high school • There was no significant interaction between current structural stigma and rejection sensitivity

Nearly all (*n* = 9) studies focused on sexual minority people. Of these, six reported self-identified sex (i.e., male, female) or gender (i.e., man, woman) without reporting whether participants were cisgender. Two of these nine studies explicitly excluded participants who were not cisgender and one study included cisgender and transgender participants, but the sample was 99.2% cisgender. Of these nine studies, two focused on SMW, two focused on sexual minority men (SMM), and five included SMW and SMM. Of the studies that included both SMW and SMM, four examined gender differences. Four studies included a heterosexual comparison group and three studies examined differences between sexual minority subgroups (e.g., lesbian and bisexual). Overall, sexual identities beyond lesbian and gay were underrepresented, and sexual minority samples were very small compared to heterosexual comparison groups (see Table 3). Only two studies focused on transgender participants.

All 11 studies assessed structural stigma at the state level, with some variation in specific measures. The most common measure, used in six studies, was Movement Advancement Project's (MAP) measure of state policy environment, which is an index based on the number of protective and discriminatory SGM-related laws in each state. Similar to the MAP index, the Human Rights Campaign State Equality Index is based on protective and discriminatory state laws; this index was used by two studies. Greene et al. (2020) used both MAP and Human Rights Campaign indices and found complete agreement between the two. Two studies operationalized structural stigma as state-level laws permitting or banning civil union or marriage between two people of the same sex/gender. One study used a structural stigma index based on z-transformed scores of state-level LGB policies and state-level public attitudes toward LGB people. One study operationalized structural stigma as the presence of a state-level employment nondiscrimination law and state-level inclusion of gender identity or transgender status in hate crime laws; this was also the only study to use a city-level measure—the Municipality Equality Index. Finally, only one study investigated more than one level of minority stressors or stigma: Pachankis et al. (2014) examined whether rejection sensitivity (individual-level) interacted with structural stigma.

Studies varied widely in their use of alcohol outcome measures (see Table 3). Four studies used clinical measures for AUD, including the *DSM-5* AUD, the Alcohol Use Disorders Identification Test-Consumption (AUDIT-C), an ICD-9 code, and the Alcohol Use Disorder and Associated Disabilities Interview Schedule-5 (AUDADIS-5). Five studies used measures of binge drinking, heavy episodic drinking (HED), or high intensity binge drinking. Additional alcohol outcome measures include average number of alcoholic drinks per day, alcohol use (versus non-use), and hazardous drinking (i.e., HED, intoxication, adverse drinking consequences, and alcohol dependence symptoms).

### 3.2 Methodological quality

The methodological quality of included studies was high. Seven studies met all eight criteria for methodological quality (see Table 1) using the JBI cross-sectional studies checklist (Joanna Briggs Institute, 2020). The remaining four studies (du Bois et al., 2018; Greene et al., 2021; Greene et al., 2020; Pachankis et al., 2014) met all but one criterion. Two of these studies did not meet the criterion, “Were the outcomes measured in a valuable and reliable way” because they used alcohol measures that were not based on clinical criteria or established guidelines and were not previously validated (both measures used a single item; du Bois et al., 2018; Pachankis et al., 2014). The other two studies did not meet the criterion, “Were the study subjects and the setting described in detail?” – one did not include absolute numbers or percentages for age, education, or income (Greene et al., 2021), and neither study included absolute numbers or percentages for race or ethnicity (Greene et al., 2021, 2020). However, both studies used BRFSS data, which is a large probability sample of the U.S. non-institutionalized population, so the risk of bias remains low. JBI checklists are not intended for interpretation as scales with total scores. Although scores have been calculated as a reference, they should not be misconstrued as an overall rating (see Table 1).

### 3.3 Structural stigma and alcohol use disorder

Four studies examined the association between structural stigma and AUD. There was overall moderate support for a positive association. In a large national sample, Hatzenbuehler et al. (2010) found that both heterosexual and LGB adults had higher odds of AUD after passage of a same-sex marriage ban, compared to odds before the ban, with greater magnitude among LGB than heterosexual adults. Similarly, in an online study with SMM, anti-LGBTQ policy environment was positively associated with AUD, and anti-LGBTQ policy environment strengthened the positive association between structural racism and AUD among Black SMM (English et al., 2021). In the same study, neither anti-LGBTQ policy environment, nor structural racism, nor the interaction between the two, were significantly associated among White SMM (English et al., 2021). In a large national sample, Drabble et al. (2021) found no significant difference in probability of AUD among either SMW or SMM in states with comprehensive LGBTQ+ policy protections compared to those in states without comprehensive protections. Additionally, in a study of transgender veterans, there was no association between state-level inclusive hate crime laws or employment nondiscrimination laws and AUD (Blosnich et al., 2016). Further, opposite the hypothesized direction, veterans with AUD tended to live in cities with higher city-level LGTBQ equality, and higher city-level LGTBQ equality was associated with higher odds of AUD; however, the effect was very small (Blosnich et al., 2016).

### 3.4 Structural stigma and heavy episodic drinking

Six studies investigated associations between structural stigma and HED; findings provided moderate to strong evidence of a positive association, especially among SMW. In a national online study, comprehensive LGBTQ+ policy protections were significantly associated with fewer days of HED among SMW, particularly bisexual women (Drabble et al., 2022). Further, in a large national sample, Greene et al. (2020) found that lesbian women in states without LGBTQ+ inclusive statutes had higher odds of binge drinking than straight (heterosexual) women, whereas odds did not significantly differ in states with inclusive statutes (i.e., the disparity was only significant in more hostile policy environments). Bisexual women in this study had higher odds of binge drinking than straight women in all states, but this disparity was substantially smaller in states with an inclusive policy environment. In another study using the same dataset, Greene et al. (2021) found that in states with inclusive statutes, a stricter alcohol policy environment was associated with lower odds of binge drinking among women, but there was no association in states without inclusive statutes, suggesting that stricter alcohol policies may be protective against binge drinking among women, but only in LGBTQ+ inclusive policy environments (Greene et al., 2021). Similarly, in a subsample from a national study, structural stigma was positively associated with binge drinking among lesbian women (Manser and Du Bois, 2021).

Two studies had mixed or null findings among SMW. One study utilized a quasi-experimental design to compare SMW in a community sample who were interviewed at various points throughout the legislation process of passing a state bill permitting civil unions (i.e., before bill signing, after signing but pre-enactment, or after enactment; Everett et al., 2016). SMW with high school education or less reported lower HED after the bill was signed compared to those interviewed before; however, enactment of this legislation was associated with higher HED among the full sample (Everett et al., 2016). Similarly, in a large national sample, comprehensive LGBTQ+ protections were not associated with either odds or probability of high intensity (8+ drinks/day) drinking among SMW or heterosexual women (Drabble et al., 2021).

There was some evidence of a positive association between structural stigma and HED among SMM. In a large national sample, comprehensive LGBTQ+ protections were associated with lower odds of high intensity (8+ drinks/day) drinking among SMM, but not among heterosexual men (Drabble et al., 2021). Additionally, in states with comprehensive LGBTQ+ protections, SMM had lower probability of high intensity drinking than SMM in states without such protections (Drabble et al., 2021). However, some studies had null findings among SMM. In a large national sample, there was no significant interaction between sexual identity and inclusive policy environment for gay and bisexual men (Greene et al., 2020). In another study using the same dataset, there were no significant associations between sexual identity, alcohol policy environment, presence of inclusive statutes, and binge drinking among men (Greene et al., 2021). Similarly, in a subsample from a national study, structural stigma was not associated with binge drinking among gay men (Manser and Du Bois, 2021).

### 3.5 Structural stigma and additional alcohol measures

Three studies examined the association between structural stigma and alcohol-related outcomes other than AUD or HED; there was overall moderate to strong support for a positive association with these outcomes. In a community sample of SMW, the signing and enactment of state-level legislation allowing same-sex civil unions were associated with fewer adverse drinking consequences, but not alcohol dependence symptoms or intoxication (Everett et al., 2016). In a subsample of transgender adults from a national study, a more inclusive policy environment was associated with a decrease in average number of alcoholic drinks per day (du Bois et al., 2018). In an online sample of SMM university students, there was no association between past (i.e., during high school) or current structural stigma and alcohol use; however, past structural stigma strengthened the association between rejection sensitivity and more frequent alcohol use (Pachankis et al., 2014).

### 3.6 Racial/ethnic differences

Only two studies examined racial/ethnic differences in the association between structural stigma and negative alcohol-related outcomes among SGM people and findings were mixed. In one study, structural stigma was positively associated with AUD among Black SMM, but not White SMM (English et al., 2021). Further, anti-LGBTQ policy environment strengthened the positive association between structural racism and AUD among Black SMM (English et al., 2021). In contrast, the second study found that White SMW reported more alcohol dependence symptoms after legislation permitting same-sex civil unions was signed compared to White SMW interviewed prior to bill signing, but Black and Latina SMW did not. (Everett et al., 2016). Further, after the bill was signed, Black and Latina SMW reported less frequent intoxication than White SMW (Everett et al., 2016).

## 4 Discussion

This review fills a gap by summarizing and synthesizing the small but growing body of literature on the relationship between structural stigma and alcohol use among SGM people, which to our knowledge has not been done previously. Findings provided moderate to strong support for a positive association between structural stigma and negative alcohol-related outcomes including AUD, HED, and adverse drinking consequences. This association was more pronounced among SMW than SMM, and among bisexual women than lesbian women. Overall, findings indicate that approaches to reducing poor alcohol-related outcomes and inequities among SGM people will likely be more effective if they address structural stigma.

Subgroup differences in the positive association between structural stigma and poor alcohol-related outcomes among sexual minority people are consistent with prior research documenting variation in alcohol outcomes by sexual identity and gender. In general, associations between structural stigma and alcohol use were stronger among SMW than among SMM (Greene et al., 2020, 2021; Manser and Du Bois, 2021); this is unsurprising given previous findings that alcohol-use disparities are more pronounced among SMW than SMM (Hughes et al., 2016, 2020; McCabe et al., 2019). Further, findings of stronger associations between structural stigma and alcohol use among bisexual women than lesbian women (Drabble et al., 2022; Greene et al., 2020, 2021) are in line with previous findings that bisexual women experience worse alcohol-related outcomes than lesbian women (Hughes et al., 2020; Schuler and Collins, 2020). However, few studies in this review examined differences by gender or sexual identity.

Some findings suggest that associations between structural stigma and negative alcohol-related outcomes may differ by race or ethnicity, but overall, relevant findings were mixed. English et al. (2021) found that structural stigma was more strongly associated with AUD among Black SMM than White SMM, and that it amplified the positive association between structural racism and AUD. This echoes previous findings that Black sexual minority people may experience worse alcohol-related outcomes than their White counterparts (Greene et al., 2020; Schuler et al., 2020). Drinking may serve as a coping mechanism, in particular among people who experience multiple forms of oppression (e.g., racism, homophobia, transphobia) simultaneously (Lewis et al., 2016). This may be related to the harm of multiple interlocking systems of oppression (e.g., sexism, racism, homophobia, class oppression; Collins, 1991; Crenshaw, 1989, 1991, 1995; The Combahee River Collective, 1977) on health, above and beyond a sum of its parts (Bowleg, 2008, 2012). In contrast, however, Everett et al. (2016) found an unexpected positive association between protective legislation and greater alcohol dependence symptoms among White SMW, but not among Black or Latina SMW. Given that findings were mixed and given the small number of studies that examined racial or ethnic differences, caution is warranted in interpretation of these findings. Future work is needed to further investigate potential racial/ethnic differences in the relationship of structural stigma and alcohol-related outcomes among SGM people, as well as the role of interlocking systems of oppression.

Only two studies examined the relationship between structural stigma and alcohol-related outcomes among gender minority people, and findings were mixed. du Bois et al. (2018) found that a more inclusive policy environment was associated with lower alcohol use among transgender adults. In contrast, Blosnich et al. (2016) found the opposite; however, the effect was very small, and could potentially be explained by endogeneity. Additionally, this study included patients who had an ICD-9 diagnosis of gender identity disorder, so those who concealed their minority gender identity would have been missed (Blosnich et al., 2016). Previous studies have shown that lower structural stigma is associated with lower odds of avoiding healthcare due to fear of mistreatment among transgender people (Goldenberg et al., 2020). It is possible that people in higher equality environments were more likely to disclose gender identity and to seek AUD treatment when needed, thereby creating a spurious positive association between high equality and greater odds of AUD. It is also possible that higher equality fosters connectedness to the SGM community, which has been associated with higher substance use in previous studies (Demant et al., 2018; Feinstein et al., 2017), possibly due to more permissive alcohol norms (Cochran et al., 2012) or perceived permissive alcohol norms (Boyle et al., 2020) among sexual minority people, although it is unclear if this is also the case for gender minority adults. Finally, AUD may be too high of a threshold, leading to potential misclassification.

Although findings overall were supportive of a moderate to strong association between structural stigma and poorer alcohol-related outcomes among SGM people, there were also some unexpected findings. For example, Everett et al. (2016) found that signing and enactment of a bill permitting same-sex civil unions were associated with worse alcohol-related outcomes compared to pre-signing and pre-enactment among some SMW. However, alcohol measures were based on the past 12 months and may not have been sensitive to legislative changes that were confined to a four-month period. Further, changes in alcohol use in response to legislation may not have been immediate enough to be reflected in the study findings.

The body of literature reviewed has several strengths and limitations that should inform future research. Most studies used national samples or represented a majority of U.S. states and regions. Additionally, most studies had large sample sizes. Studies were also of high methodological quality. However, nearly half used non-probability samples and all studies were cross-sectional; research with probability samples and longitudinal designs is needed to capture periods of policy change. Additionally, even among studies using large national samples, samples of SGM participants were relatively small, making it less likely they were representative, despite probability sampling. Studies underrepresented transgender and nonbinary people, SGM people of color, and sexual identity subgroups beyond gay and lesbian (e.g., bisexual, pansexual, queer). Further, even among studies with diverse samples, few examined subgroup differences by race, ethnicity, gender, or sexual identity, and none examined age group differences. Researchers should strive to recruit samples that are representative of the diversity of SGM people and consider oversampling of smaller groups that may not otherwise be adequately represented. Given prior research that shows subgroup differences (e.g., by race, ethnicity, sexual identity, gender identity) in the relationship between stigma and alcohol use at the individual and interpersonal levels, studies are needed that investigate such differences in associations between structural stigma and alcohol use.

There were also potential alcohol measurement concerns in the included studies. In particular, alcohol measures were heterogeneous and ranged from a very low threshold, such as whether a participant drank any amount of alcohol each day (Pachankis et al., 2014), to a high threshold, such as meeting diagnostic criteria for AUD (Blosnich et al., 2016; Drabble et al., 2021; English et al., 2021; Hatzenbuehler et al., 2010). Although low threshold and high threshold measures are appropriate for certain research questions, a low bar may not meaningfully differentiate participants who drink enough to harm health, and a high bar may miss participants whose use is harmful to their health but falls short of AUD. Given that previous research has found that permissive cultural norms and perceived norms are associated with heavier alcohol use among sexual minority people (Boyle et al., 2020; Cochran et al., 2012), it may be especially important to design studies that capture whether structural stigma is associated with heavier alcohol use that may not reach AUD criteria.

This review illuminates the need for expanded measures of structural stigma in future research. Only one study examined public opinion or social stigma (Pachankis et al., 2014), despite it being both a potential cause and effect of anti-SGM policies, and only one study examined city-level structural stigma (Blosnich et al., 2016). Future research should investigate the influence of public opinion and social stigma on SGM alcohol use. Future research should also examine whether more proximal measures of structural stigma at institutional, neighborhood, and regional levels are associated with alcohol use among SGM people, and whether proximal measures are equally, or more important, than more distal measures of stigma at the state or national levels. Additionally, most included studies utilized an index of multiple laws and policies to measure structural stigma, which provides a picture of how the overall policy environment influences alcohol use among SGM people. However, future research should examine which specific policies and policy types are most impactful to help prioritize intervention and advocacy targets.

Findings in this review point to several important gaps in the literature with implications for future research. First, there was an overall lack of multilevel (i.e., individual, interpersonal, and structural) stigma research; only one study examined more than one level simultaneously (Pachankis et al., 2014). It is important to understand how the different levels influence alcohol use among SGM people, both alone and synergistically, to inform interventions that adequately address the harms of multilevel stigma. Second, no studies examined how resiliency factors (e.g., LGBTQ community connectedness) may buffer against structural stigma. Future studies should aim to identify resiliency factors to inform intervention strategies to mitigate the harms of structural stigma. Third, due to numerous underrepresented groups as mentioned previously, greater diversity (e.g., by race, ethnicity, sexual identity subgroup, gender identity subgroup) of SGM samples would enhance generalizability of the findings. Due to the rapid escalation of anti-SGM legislation in recent years aimed at gender minority people in particular (e.g., criminalization of gender-affirming care for transgender people of all ages), the need for future research on structural stigma and alcohol use among this population is especially critical (Movement Advancement Project, 2023a, 2023b). Relatedly, data collection on SGM identity in health care and on national surveys in line with current recommendations (e.g.., expansive sexual identity and gender identity response options) is crucial to making progress in stigma-related research (National Academies of Sciences Engineering and Medicine, 2020) by improving availability of datasets with large, diverse samples.

### 4.1 Limitations of this review

This review, despite filling a gap in the literature on structural stigma and alcohol-related outcomes among SGM people, is not without limitations. It included peer-reviewed journal articles only. Because journals are more likely to publish significant findings, there is a potential for publication bias. Additionally, despite a comprehensive search strategy, it is possible that we did not identify all studies meeting inclusion criteria. In particular, authors do not always use the term “structural stigma” to describe measures that meet its definition and there are many possible ways to operationalize this construct, making it difficult to capture all articles that meet criteria. It is also possible that we missed relevant articles published prior to 2010. Finally, by restricting our search to studies conducted in the U.S., this may limit generalizability of findings to other countries, particularly those with very different sociopolitical contexts.

### 4.2 Implications for policy and practice

This review highlights the importance of reducing structural stigma through policy change, both by eliminating laws that are hostile to SGM people and by enacting laws that are protective. Stigma is a fundamental cause of population health inequities (Hatzenbuehler et al., 2013) such as alcohol-related health inequities among SGM people. Even as treatment of diseases improves over time, health inequities persist due to underlying social and structural factors (Link and Phelan, 1995; Phelan et al., 2010). If health professionals and policy makers do not address underlying structural issues, efforts to effectively address alcohol-related disparities and inequities will be impeded (Hatzenbuehler et al., 2013; Link and Phelan, 2001). Examples of hostile policies that should be eliminated include, but are not limited to, Don't Say Gay or Trans laws (i.e., laws that prohibit discussion of sexual or gender identity in public schools), gay and trans panic laws (i.e., laws that excuse violence, including murder, toward SGM people as justifiable “panic”), or religious exemption laws (e.g., government officials refusing marriage licenses to SGM people for religious reasons). Example protective laws include housing or employment non-discrimination laws that explicitly enumerate sexual identity and gender identity as protected classes, or bans on conversion therapy.

Even in protective policy environments, individual- and interpersonal-level interventions are needed. Enactment of a protective policy does not necessarily mean that policy will be implemented and/or enforced or that it will influence the social environment (e.g., interactions with neighbors, family, strangers), particularly in the short term. Further, the public discourse (e.g., anti-SGM ad campaigns) around an SGM-related ballot initiative may be harmful, regardless of whether the bill passes (Maisel and Fingerhut, 2011). Additionally, due to a long history of stigma and a hostile sociopolitical environment, changing policies may not undo harm or improve alcohol-related outcomes in the short term. Therefore, individual- and interpersonal-level interventions, such as SGM-tailored alcohol treatment, are important. Further, given the increasingly hostile policy landscape for gender minority people in particular, individual- and interpersonal-level interventions tailored for gender minority people, in addition to policy change, may be especially crucial. A recent study found that a decrease in structural stigma was longitudinally associated with an increase in SGM-tailored programming being offered at substance use treatment facilities (Cascalheira et al., 2022), thereby illustrating the potential of multilevel approaches in addressing alcohol-related health inequities among SGM people. Finally, given the impact of structural stigma on alcohol use among SGM people, clinicians need to be aware of these inequities, incorporate conversations about alcohol use into care for SGM people, and be competent in providing care to SGM people.

### 4.3 Conclusions

Findings from this review support the positive association between structural stigma and poor alcohol-related outcomes among sexual minority people, with stronger associations among SMW than among SMM, and among bisexual women in particular. Few studies included gender minority people or examined differences by race or ethnicity; further research is needed in these areas. Findings build on evidence in the existing literature that individual- and interpersonal-level stigma contribute to poor alcohol-related outcomes and inequities among SGM people and indicate that more research is needed to expand upon the small body of literature on stigma at the structural level. Findings suggest that to adequately address negative alcohol-related outcomes and inequities among SGM people, prevention and intervention efforts should focus on structural stigma as well as individual- and interpersonal-level factors.

## Author disclosures

### Role of funding source

Nothing declared.

## CRediT authorship contribution statement

**Sarah S. Zollweg:** Conceptualization, Data curation, Methodology, Formal analysis, Funding acquisition, Project administration, Writing – original draft, Writing – review & editing. **Joseph A. Belloir:** Data curation, Methodology, Formal analysis, Writing – original draft, Writing – review & editing. **Laurie A. Drabble:** Writing – original draft, Writing – review & editing. **Bethany Everett:** Writing – original draft, Writing – review & editing. **Jacquelyn Y. Taylor:** Writing – original draft, Writing – review & editing. **Tonda L. Hughes:** Writing – original draft, Writing – review & editing.

## Declaration of Competing Interest

No conflict declared.

## Figures and Tables

**Table:** 

	Terms	Results
1	"sexual and gender minorities"[MeSH Terms] OR "homosexuality"[MeSH Terms] OR "bisexuality"[MeSH Terms] OR "Transgender Persons"[Mesh] OR "Sexuality"[Mesh] OR "Gender Identity"[Mesh] OR bicurious OR bisexual OR bisexuals OR bisexuality OR GLB OR GLBQ OR GLBs OR GLBT OR GLBTQ OR heteroflexible OR homosexual OR homosexuals OR homosexuality OR "mostly heterosexual" OR "non heterosexual" OR nonheterosexual OR gay OR gays OR lesbian OR lesbians OR lesbianism OR lesbigay OR LGB OR LGBQ OR LGBs OR LGBT OR LGBTQ OR queer OR "same gender loving" OR "same sex attracted" OR "same sex attraction" OR "same sex couple" OR "same sex couples" OR "same sex relations" OR "same sex relationship" OR "sexual and gender minority" OR "sexual and gender minorities" OR "gender minority" OR "gender minorities" OR "sexual minority" OR "sexual minorities" OR "sexual identity" OR "sexual identities" OR "gender identity" OR "gender identities" OR "sexual orientation" OR "sexual preference" OR "women loving women" OR "women who have sex with women" OR WSW OR "men who have sex with men" OR MSM OR "men loving men" OR genderqueer OR "gender nonconforming" OR "gender non-conforming" OR transgender OR transsexual OR nonbinary OR "non binary" OR "two spirit"	57,376
2	"Public Nondiscrimination Policies"[Mesh] OR "Public Policy"[Mesh] OR "Homophobia"[Mesh] OR "Prejudice"[Mesh] OR "Social Stigma"[Mesh] OR "Social Norms"[Mesh] OR "protective policy" OR "protective policies" OR "state policy" OR "state policies" OR "state level policy" OR "state level policies" OR "antidiscrimination policy" OR "antidiscrimination policies" OR "anti-discrimination policy" OR "anti-discrimination policies" OR "nondiscrimination policy" OR "nondiscrimination policies" OR "non-discrimination policy" OR "non-discrimination policies" OR "social policy" OR "social policies" OR "institutional policy" OR "institutional policies" OR "antidiscrimination law" OR "anti-discrimination law" OR "nondiscrimination law" OR "non-discrimination law" OR "protective law" OR "state law" OR "state level law" OR "antidiscrimination laws" OR "anti-discrimination laws" OR "nondiscrimination laws" OR "non-discrimination laws" OR "protective laws" OR "state laws" OR "state level laws" OR "marriage equality" OR "marriage ban" OR "same sex marriage" OR "gay marriage" OR "hate crime" OR "systemic exclusion" OR "structural exclusion" OR segregation OR antigay OR "anti-gay" OR homophob* OR transphob* OR "anti LGBT" OR "antiLGBT" OR "anti homosexual" OR "antihomosexual" OR antihomosexuality OR "anti-homosexuality" OR heteronorm* OR heterosexis* OR cissexis* OR cisnorm* OR prejudic* OR discrim* OR attitude* OR stigma OR "social norm" OR "social norms" OR "cultural norm" OR "cultural norms" OR "community norm" OR "community norms" OR "structural factor" OR "structural factors" OR "structural stressor" OR "structural stressors" OR structural AND "minority stress" OR structural AND "minority stressor" OR structural AND "minority stressors" OR "systemic factor" OR "systemic factors" OR "systemic stressor" OR "systemic stressors" OR "institutional stressor" OR "institutional stressors" OR "public policy" OR "public policies" OR "adoption law" OR "adoption laws" OR "adoption policy" OR "adoption policies" OR "same sex adoption"	594,647
3	"Alcohol-Induced Disorders"[Mesh] OR "Alcohol-Related Disorders"[Mesh] OR "Alcohol Drinking"[Mesh] OR "Alcoholism"[Mesh] OR alcohol* OR ethanol* OR "alcohol use disorder" OR drink* OR drunk* OR "binge drink" OR "binge drinking" OR "binge drinker" OR "binge drinkers" OR "hazardous drinking" OR liquor* OR beer* OR wine* OR intoxic* OR "alcohol-induced disorder" OR "alcohol-induced disorders" OR "alcohol-related disorder" OR "alcohol-related disorders"	401,066
4	1 + 2 + 3	917

*Note*. PubMed search performed May 18, 2022. Filter: from 01/01/2010 – 05/18/2022. Four additional permutations of this search strategy were performed in CINAHL, PsycInfo, LGBTQ+ Source, and Embase, with subject headings tailored to each database.

## Appendix A

Search Strategy
